# The probability of Lymph node metastasis with a tumor size larger than and smaller than 4 cm is different in stages T1-T3 of Siewert type II adenocarcinoma of esophagogastric junction: A Population-Based Study

**DOI:** 10.7150/jca.63392

**Published:** 2021-09-27

**Authors:** Huolun Feng, Jiabin Zheng, Chengbin Zheng, Zhenru Deng, Qianchao Liao, Junjiang Wang, Yong Li

**Affiliations:** 1 Department of gastrointestinal surgery, Guangdong Provincial People's Hospital; Guangdong Academy of Medical Sciences, Guangzhou 510080, Guangdong, P. R. China.; 2 The Second School of Clinical Medicine, Southern Medical University, Guangzhou 510515, Guangdong, P. R. China.

**Keywords:** lymph node metastasis, tumor size, adenocarcinoma of esophagogastric junction, SEER, nomogram

## Abstract

**Background:** In adenocarcinoma of esophagogastric junction (AEG), the relationship between tumor size (TS) and lymph node metastasis (LNM) is unclear. This study aimed to explore the relationship between TS and LNM, and to construct a prediction model for LNM.

**Materials and Methods:** Data from 4649 Siewert type II AEG patients were retrospectively acquired from the Surveillance, Epidemiology, and End Result (SEER) database. TS data was analyzed as a continuous variable, but also divided into 1-cm-interval categorical groups for further analysis. The logistic regression model and restricted cubic spline (RCS) model was used to explore the relationship between TS and LNM, after adjusting for covariates. Internal validations as well as external validation (Single-Center data) were used to check our LNM prediction model.

**Results:** TS and LNM showed a significant relationship in the logistic regression analysis, regardless of the TS data being entered as a continuous or a categorical variable, after adjusting for covariates. The logistic regression model and RCS consistently showed that larger TS resulted in larger Odds Ratio (OR) values. When tumors were larger than 4 cm, the OR value remained relatively constant. The receiver operator characteristic curve evaluated the nomogram by the area under the curve (AUC) (AUC=0.737, in internal validation; AUC=0.626, in external validation), and the calibration curve of the nomogram showed an improved prediction system.

**Conclusions:** In Siewert type II T1-T3 stage AEG patients, we reported that LNM increased with TS up to 4-cm, and our nomogram provided a simple tool to predict LNM.

## Introduction

Recently, the incidence of adenocarcinoma of esophagogastric junction (AEG) has increased 1-4, and research into AEG has therefore received more and more attention. AEG is defined as a tumor with an epicenter within 5 cm of the esophagogastric junction. The Siewert classification, universally accepted by many experts, divides AEG into 3 types depending on the location of the epicenter of the tumor. Siewert type II AEG includes tumors located from 1 cm above to 2 cm below the esophagogastric junction, and is often defined as a true tumor of the cardia 5, 6. In the eight edition American Joint Committee on Cancer (AJCC) cancer staging manual, Siewert type III was reclassified from the esophageal cancer staging system to the gastric cancer staging system. Siewert type II AEG on the other hand, still uses the esophageal cancer staging system where tumor size (TS) classification is neglected.

As the esophageal cancer staging system to predict Siewert type II AEG prognosis is not exact, there is much debate surrounding it 7-9. There were concerns over the low accuracy of LNM (lymph node metastasis) diagnosis, and inadequate factors included in the current staging system. The node (N) staging of AEG relies on the number of lymph node metastatic. Preoperative diagnosis of lymph node metastasis mainly relies on CT, endoscopic ultrasound, and MRI, which are mainly based on the size of the lymph nodes 9-12. The diagnostic accuracy of LNM prediction using these methods is not particularly high as the size of the lymph nodes evaluation is greatly affected by others factors, and thus is largely dependent on the physician's evaluation. Pathological diagnosis is the gold standard for judging LNM 13. However, Pathological diagnoses are easily affected by surgical approach, surgical method, lymph node dissection, and other procedures. In Siewert type II and III early AEG, studies have shown that TS is related to LNM. However, within the T1-T3 stages of Siewert type II AEG the relationship between TS and LNM has not yet been described. Additionally, TS is undervalued as a prognostic tool.

Therefore, in this study we illustrated the relation between TS and LNM, and constructed LNM prediction model that can be applied in clinical practice.

## Materials and Methods

Data were collected using the SEER*Stat software (Version 8.3.2) from Surveillance, Epidemiology, and End Result (SEER) database (2002-2016). Eligibility criterion was as follows: (1) patient diagnosed with Siewert type II AEG at 20 years old or after, (2) T stage was T1-T3 with a TS less than 30 cm, (3) metastasis (M) stage was M0, (4) patient underwent radical surgery, postoperative survival time was more than two months, and complete follow-up data was accessible, (5) included variables were also complete and accessible. SEER database selected tumor site ICD-O-3 code 160 and “CS site-specific factor 25”. Histology type coding were 8140-8147, 8160-8162, 8180-8221, 8250-8507, 8514-8551, 8571-8574, 8576, and 8940-8941 14, 15. Single-Center data were selected to validate a LNM prediction model which was based on SEER data (the selection criteria are close to the above). The SEER database is a public database, so institutional ethical approval and informed consent were not required. In the single-center cohort data (Guangdong Provincial People's Hospital), all patients signed the preoperative informed consent, patients' identification information had been removed, institutional review board approval was not required.

Data selection included age, gender, race, grade, T stage, N stage, TS, examined lymph node, survival time, and survival condition. TS was grouped at 1 cm intervals 16, 17. When larger than 7 cm, tumors were grouped together due to the small number of samples and high positive rate of lymph nodes. TS ranges were divided into the following subgroups: 1 (0-1 cm), 2 (1.1-2 cm), 3 (2.1-3 cm), 4 (3.1-4 cm), 5 (4.1-5 cm), 6 (5.1-6 cm), 7 (6.1-7 cm), and 8 (≥7.1 cm). Additionally, TS as a raw continuous variable was also analyzed.

### Statistical analysis

R software (version 3.61) was used for statistical analysis. Baseline characteristics were showed. Continuous variables are expressed as the median [IQR] and categorical variables were reported as frequencies with percentages. A χ^2^ test or Fisher test was used to compare categorical data. A Cox regression model was applied to survival difference within TS groups after adjustment other confounders. A logistic regression model was used to explore the relationship between TS and LNM. Both logistic regression and stepwise regression methods were used to construct the nomogram. The receiver operator characteristic (ROC) curve validated the discrimination power of the nomogram, and the calibration curve illustrated the prediction. All statistical tests were bilateral, and P value with less than 0.05 was considered statistically significant.

Scatter plots of TS and LNM percentages were produced. In clinical data, examined lymph node affected LNM. To exclude this bias, we constructed two logistic regression models. One model adjusted examined lymph node and other covariates to only illustrate the relationship between TS and LNM, whether TS be a continuous or categorical variable. The other model adjusted all covariates except examined lymph node in order to construct a nomogram which predicted LNM solely through preoperative variables. When evaluating TS as categorical variable, we constructed a scatter plot of TS and Odds Ratio (OR) values, and when evaluating as a continuous variable, we used a restricted cubic spline (RCS) model. Internal validations as well as external validation were used to check our LNM prediction model. Subgroups were analyzed to rule out bias from less than 15 lymph nodes retrieval resulting that their node stage were not accuracy. Subgroup analysis was comparison of ROC curves corresponding to logistic models that adjustment for all confounding variables including or excluding lymph nodes retrieval was analyzed simultaneously.

## Results

**Figure 1:**
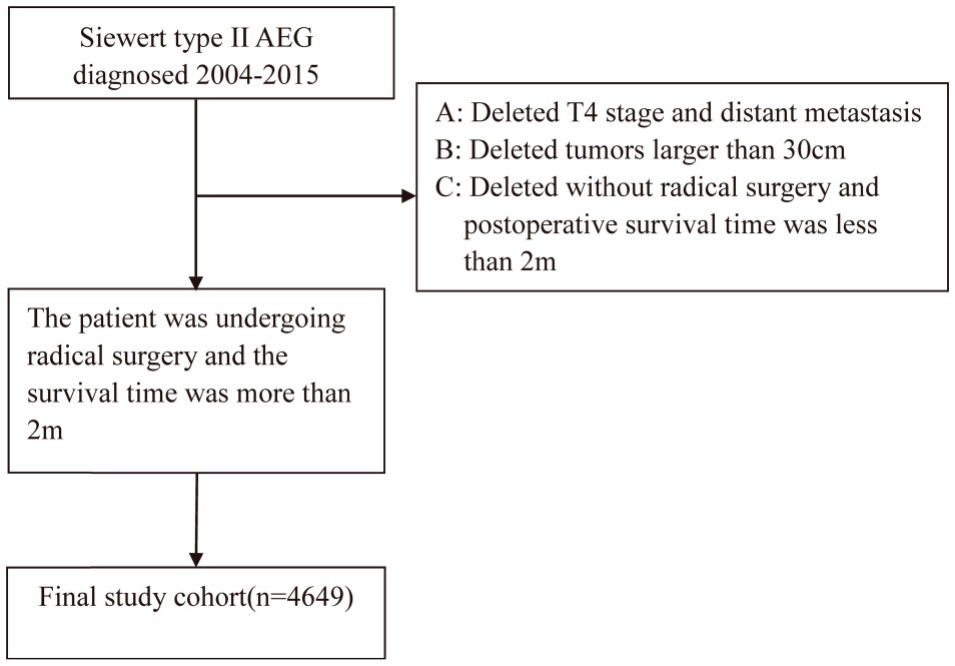
Research flowchart.

In the patient selection process, as illustrated in Figure 1, a total of 4649 patients were selected for the final study cohort. The median survival time was 39.0 months with a range of 36.6-41.4 months. The median follow-up time was 79.0 months with a range of 76.0 to 80.2 months, and the 5-year survival rate was 40.3%. Missing cases were removed from our study. The final baseline table is displayed (Table 1). The median examined lymph node was 15.

**Figure 2:**
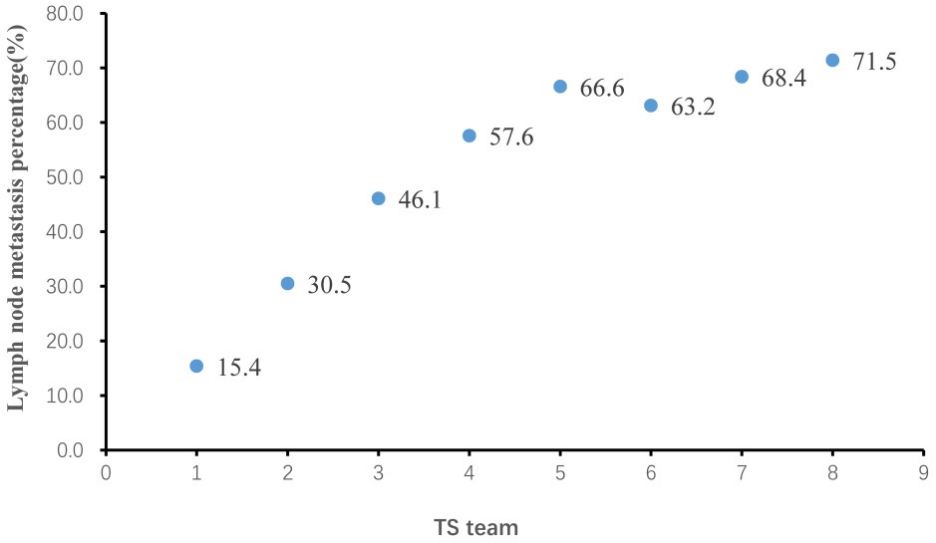
The relation of tumor size (TS) and lymph node metastasis percentage (LNMP).

**Figure 3:**
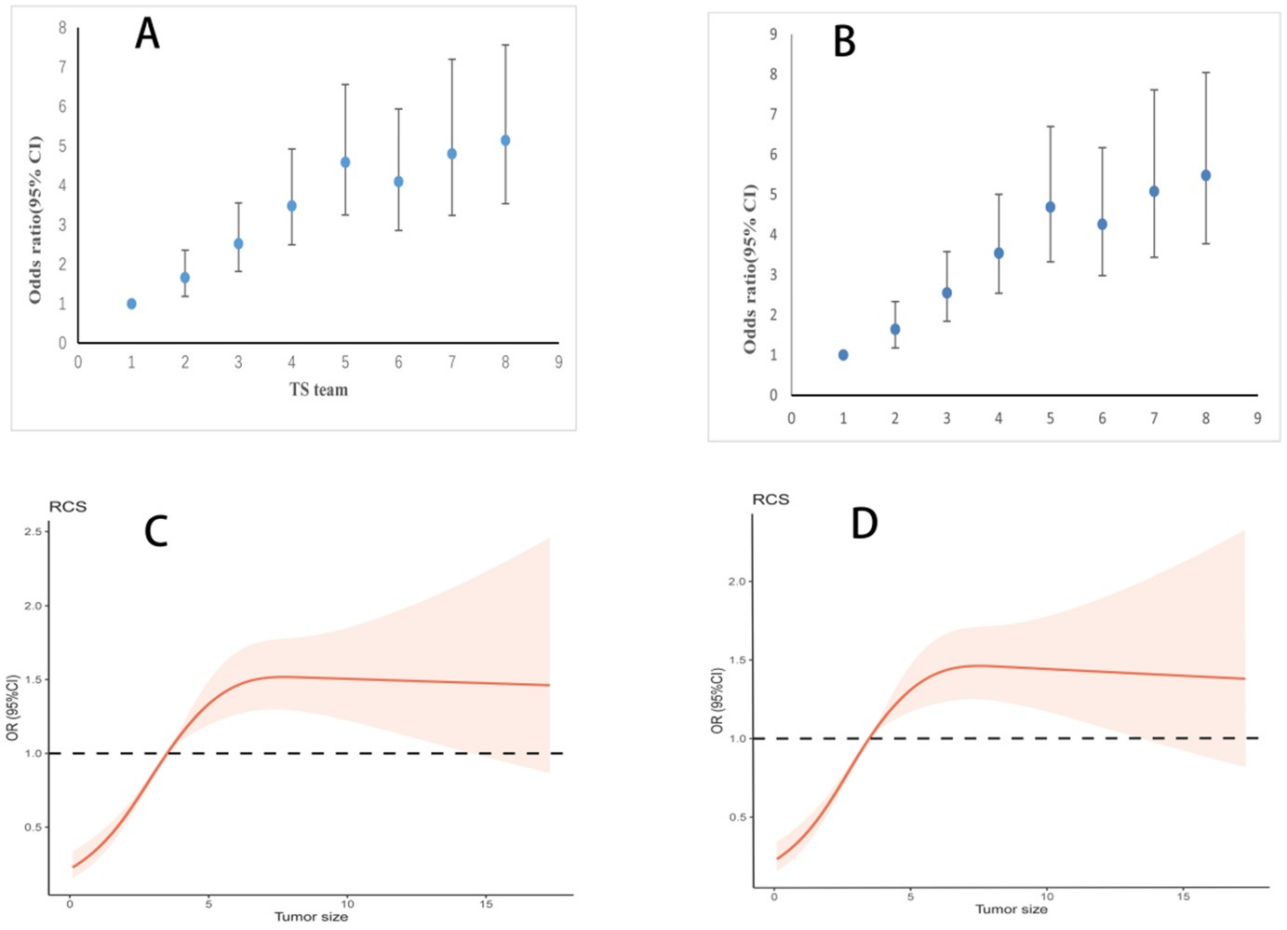
The relation of tumor size (TS) and lymph node metastasis (LNM) after adjustment for covariates.** A.** Odds ratio (95% CI) for LNM at different TS in logistic models after adjustment. Adjusted examined lymph node, age, sex, race, T stage and grade. **B.** Odds ratio (95% CI) for LNM at different TS in logistic models after adjustment. Adjusted age, sex, race, T stage and grade. **C.** Association of TS with LNM in logistics models with RCS after adjustment (4 cm reference, P for overall < 0.001, P for non-linearity < 0.001). Adjusted examined lymph node, age, sex, race, T stage and grade. TS was as continuous variable. **D.** Association of TS with LNM in logistics models with RCS after adjustment (4 cm reference, P for overall < 0.001, P for non-linearity < 0.001). Adjusted age, sex, race, T stage and grade. TS was as continuous variable.

Among all patients included in this study, TS ranged from 0.1 cm to 30 cm, with a median of 3.6 cm. Lymph node metastasis percentage (LNMP) increased with greater TS (Figure 2), LNMP was 51.7% in overall patients. However, we found that LNMP in group 5 was 66.6%, LNMP in group 6 was 63.2%, and LNMP in group 7 was 68.4%, therefore this relationship is only present up to a TS of 4 cm, that of group 5. Beyond 4 cm, there was a weak relationship between TS and LNMP. The adjusted logistic regression, including examined lymph node, also showed that larger TS coincided with greater OR values, up to a TS of 4 cm (Table 2). The scatter plot showing the relationship between LNM and OR values, and the relationship of TS, as continuous variables, and OR values, as examined by the RCS model, are clearly depicted (Figure 3A; Figure 3C). In Table 2, association of TS with overall survival was statistically significant after controlling confounders. P for trend also indicated that in each group of TS were differences of LNM between them.

The adjusted logistic regression, excluding examined lymph node, produced similar results as the regression including examined lymph node (Figure 3B). Meanwhile, TS as continuous variable, following adjustment excluding examined lymph node, also showed parallel findings (Figure 3D). In subgroup analysis, different ROC curve was near each other (overall: including lymph nodes retrieval, AUC= 0.742, excluding examined lymph node, AUC=0.737; less than 15 lymph nodes retrieval: including lymph nodes retrieval, AUC= 0.748, excluding examined lymph node, AUC=0.739; more than 15 lymph nodes retrieval: including lymph nodes retrieval, AUC= 0.733, excluding examined lymph node, AUC=0.733). Combined with this result, we can basically reduce bias due to insufficient examined lymph node.

**Figure 4:**
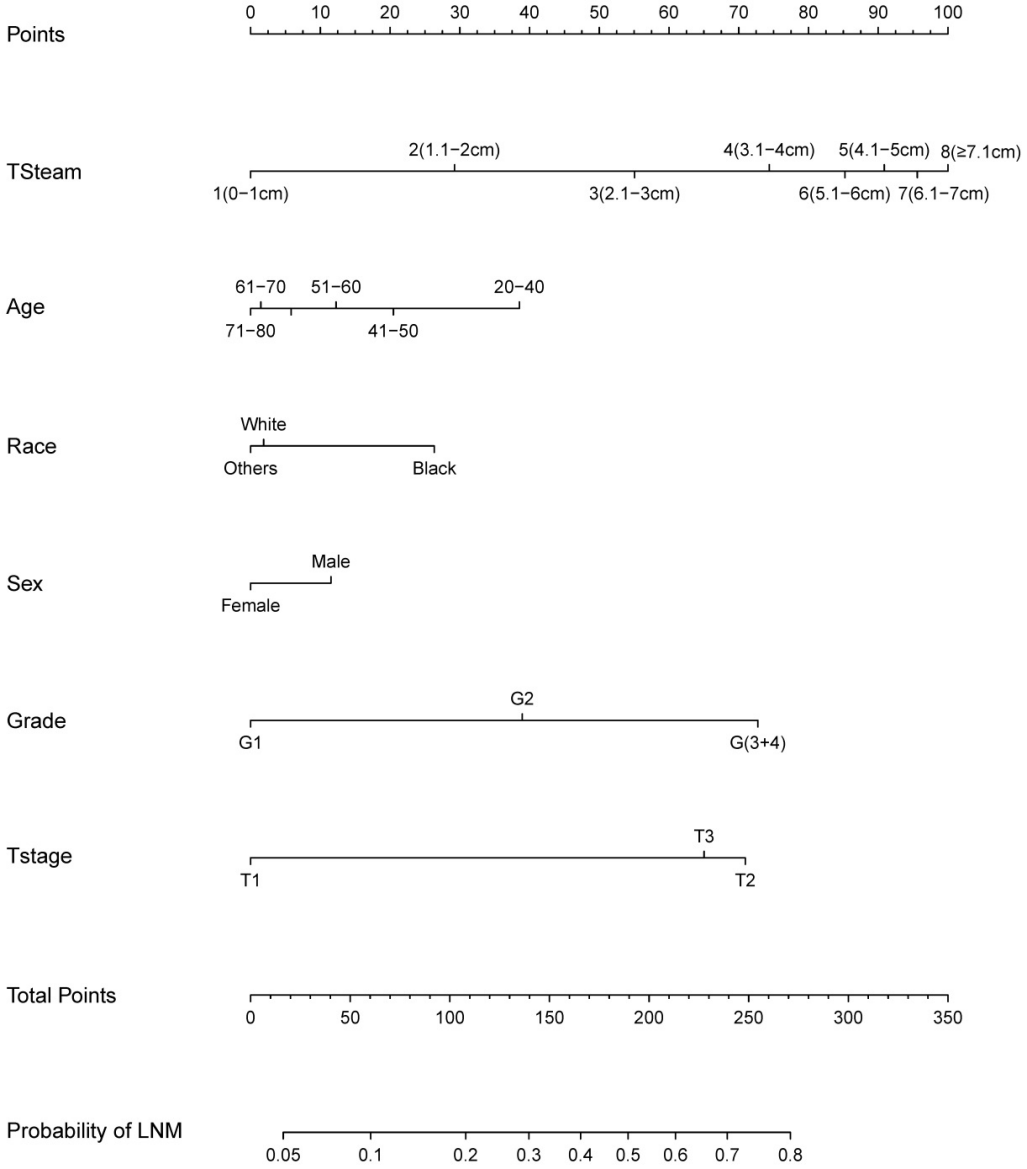
Nomogram for predicting lymph node metastasis (LNM).

**Figure 5:**
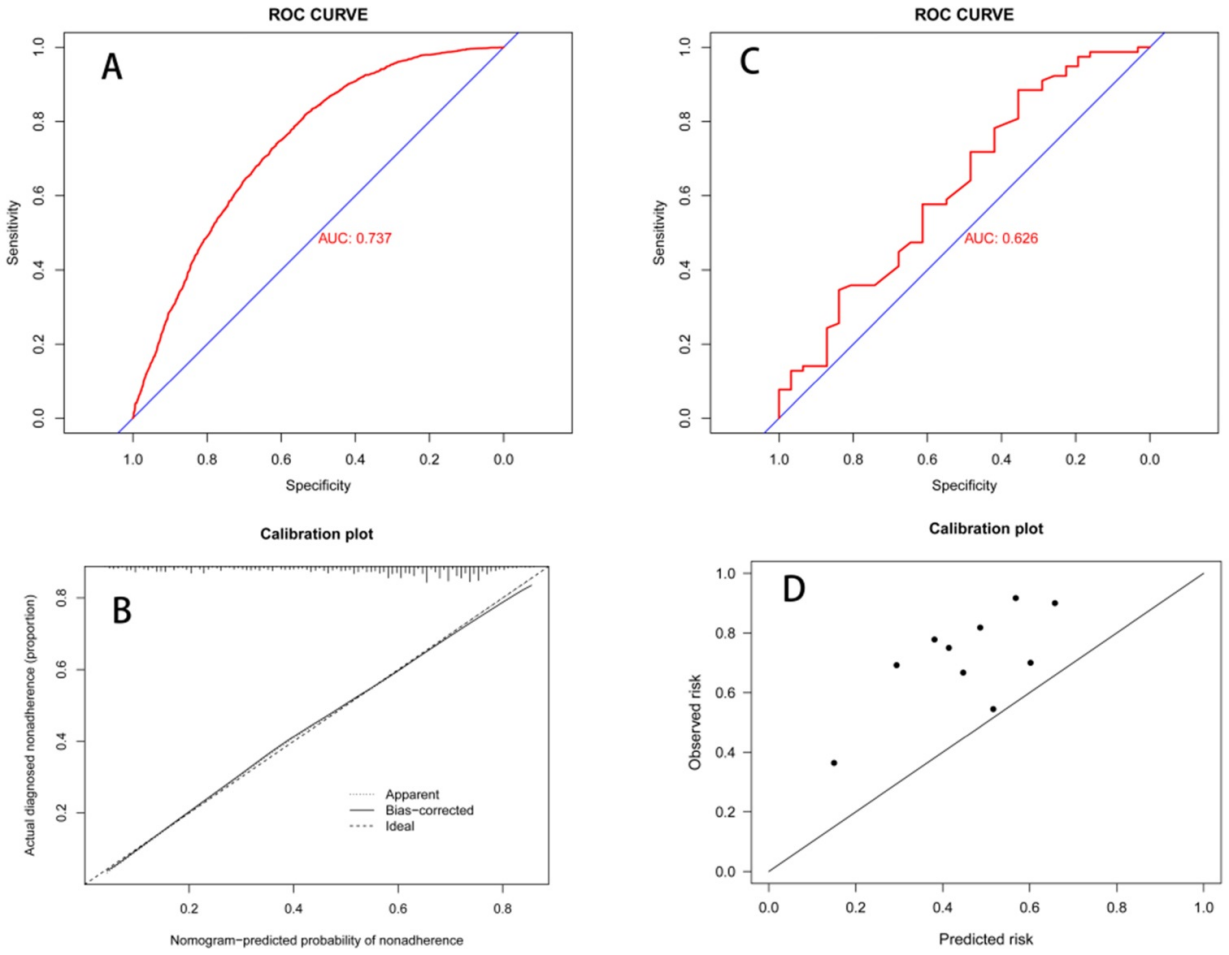
** A.** Receiver operating characteristic (ROC) curves for the nomogram in SEER data (AUC = 0.737). **B.** Calibration plot for the nomogram in SEER data by Bootstrap method. **C.** ROC curves for the nomogram in Single-Center data (AUC = 0.626). **D.** Calibration plot for the nomogram in Single-Center data.

Based on the logistic regression model, we constructed a nomogram to predict LNM (Figure 4). The nomogram showed that TS had the largest effect on LNM, followed by T stage, grade, age, gender, and ethnicity. Each variable on the nomogram corresponds to a particular scale, represented by a specific point value. By calculating the sum of the scores of each variable and the corresponding scale, the probability of LNM can be calculated. The ROC curve (AUC = 0.737) and the calibration curve indicated that the nomogram prediction was accuracy (Figure 5A; Figure 5C).

A total of 109 patients in our center' data was involved to only test the LNM prediction model, and LNMP was 71.6%. The baseline characteristics were showed in Table 3. Due to limited information, some variables were combined and displayed. In our center's data, the ROC curve (AUC = 0.613) showed the well discrimination performance of LNM prediction model, and the calibration curves in the single-center data show a near predicted trend (Figure 5B; Figure 5D).

## Discussion

With rising incidence rates, AEG is gradually gaining recognition 1, 3. The Japanese gastric cancer guide regards 4 cm as the threshold for the esophagogastric junction (EGJ) tumors to suffice direct surgical treatment, mainly based on the relationship between TS and LNM. This is the fact that when TS is larger than 4 cm, the EGJ line is unrecognizable. The larger TS had a greater the probability of LNM. This relationship has been confirmed in early-stage AEG 18. However, for T1-T3 stages, the relationship between TS and LNM in Siewert type II AEG was unknown. To our knowledge, this study is to define the relationship between the TS and LNM in T1-T3 stage Siewert type II AEG, based on SEER data.

TS is a risk factor that has clearly been shown to greatly affect the prognosis and recurrence of liver cancer, thyroid cancer, breast cancer, lung cancer, and others 19-23. In gastric cancer, TS was not only related to prognosis, but also LNM and the depth of invasion, which provides a reference to guide and narrow down treatment options 16, 17. This indicates that TS is of great significance when guiding clinical diagnoses and treatments in various cancers, however, regrettably, there are a limited number of studies that have illustrated the relationship between the TS and LNM, prognosis, and treatment methods, in AEG. Fang et al. 24 retrospectively analyzed the pathological data of 180 Siewert type II and III patients with AEG. Their results found that TS was an independent risk factor, and they believed that larger TS worsened prognosis due to the greater likelihood of a deeper tumor invasion. In the study, TS was grouped as 5 cm without explanation, and thus the basis for this grouping was ambiguous. Hoshino et al 25 retrospectively analyzed 48 patients with Siewert type II EGJ cancers and found that the EGJ line could not be clearly distinguished with a TS greater than 4 cm. Moreover, their results showed that in patients with TS greater than 4 cm, high lymph metastasis rates (68.8% vs 43.8%) were present, which is consistent with our findings where LNM rates exceed 50% when TS was greater than 4 cm. However, this study once again did not clarify whether TS was correlated to LNM. Regardless of the relationship between the TS and LNM or prognosis, some studies 24, 25 found a cutoff value through the ROC curve or the median. Although, examining this relationship when using the continuous variable of TS is linear by default without considering non-linear relationships, and therefore the cutoff values do not truly reflect the relationship between the TS and LNM or prognosis.

In our study, TS was grouped in 1-cm intervals 17, 26, which can more accurately reflect the relationship between TS and LNM. With an interval less than 1 cm, not only is there an increased chance of measurement error, but there is also substantially less clinical application. The use of a large sample size enabled our study to detect that LNM rates increased with tumor size, up to 4 cm. Like findings reported in gastric and esophageal cancers, we reported a strong relationship between the TS and LNM in Siewert type II AEG, deduced from the relationship between the OR values and LNM 13, 17, 18, 27 (Figure 2; Figure 3). LNMP in group 6 was 63.2%, lower than that of the two groups with tumors smaller as well as greater than 5.1 to 6 cm. A possible reason for this is that when TS was larger than 4 cm, its effect on LNM was already at a high level and tended to be stable from this level onwards. Secondly, when esophageal invasion increased beyond 4 cm, mediastinal LNM may have increased instead. We suggested that in Siewert type II AEG tumors larger than 4 cm, the LNM pattern changed from an abdominal LNM to a mediastinal LNM. Due to different LNM patterns, the examined lymph node was considered to mainly mediastinal, and acted in different surgeon. TS, when examined as a continuous variable, had similar results, further proving this relation. When smaller than 4 cm, increasing TS was strongly related to LNM, and beyond this cutoff, the LNM did not increase further. Having illustrated this relationship between TS and LNM, TS could now be used to guide D2 lymphadenectomy and improve the accuracy of the clinical N stage.

The TNM stage of AEG has been changed in the eighth edition AJCC cancer staging manual. Siewert type II was classified used the staging of esophageal cancer, however, TS is not included in this staging, or even in the staging of gastric cancer 6-8. Some studies 16, 17, 27-29 have already shown that incorporating TS into the TNM staging of gastric cancer can improve the discrimination of the staging system. These studies reported a relationship between the TS and LNM, which supports our construction of a nomogram to predict LNM from TS. Due to the high the risk of LNM in AEG patients, we suggest that the role of TS should not be ignored and instead can be of great value during the assessment of tumors and when designing treatment strategies. TNM staging could greatly benefit from including TS to further improve the predictive ability and clinical utility. In our study, T4 stage Siewert type II AEG was excluded. In the esophageal TNM staging system, T4 tumors are defined as tumors that have broken through the fibrous membrane, while in the gastric cancer TNM staging system, it is defined as tumors that have broken through the serosa. We used the esophageal cancer staging system. Based on the gastric TNM staging system, if the tumor had broken through the serosa, it would be very difficult to define the T stage. Furthermore, there is already a high degree of lymph node tumors in T4 stage Siewert type II AEG.

A reliable nomogram would greatly improve the accuracy of N staging, allow for a more accurate prognosis prediction, and guide intraoperative treatments. Analysis using ROC and the calibration curve showed an improved prediction system of our nomogram. However, if the data incorporated in this study had obtained more accurate values of tumor invasion depth, differentiation degree, and TS before surgery, the clinical application value of our nomogram would have been greatly improved. At present, computed tomography and endoscopic ultrasound are commonly used to diagnose LNM prior to surgery. However, the sensitivity of computed tomography is low, and endoscopic ultrasound is not useful for distant lymph nodes. Furthermore, preoperative examination surveys lymph nodes based on lymph node size, which is in fact interfered from adhesion of the lymph node to surrounding tissues and the degree of inflammation 10, 11, 30. In general, clinical N staging currently lacks a more effective and practical detection method. Easily obtained data was used to build a LNM prediction model, and its accuracy was not affected by inflammatory status or lymph node size. As exact TS can be detected by endoscopic ultrasound imaging, it is unaffected by local inflammation and surrounding tissues. Many studies 11, 31, 32 have shown that endoscopic ultrasound has an advantage in diagnosing the depth of Siewert type II AEG invasion, however, this method cannot distinguish between T1a and T1b stages. This accuracy is clearly enough to identify the depth of Siewert type II AEG invasion.

Patients commonly undergo routine endoscopic biopsy to confirm the tumor and grade of the tumor. However, it is entirely possible to obtain tumor invasion depth, grade, and TS data before surgery, which allows for the incorporation of this nomogram to predict LNM, and further improve the predictive accuracy of LNM by imaging examination. Combined with LNM prediction, neoadjuvant therapy should be performed before surgery, and lymph nodes should be actively cleaned during surgery. We recommend that routine endoscopic ultrasound and biopsy should be performed before surgery, so that TS can be effectively predicted before surgery to improve the prognosis of patients.

A limitation of this study is that it solely elaborated on the relationship between TS and lymph nodes, and the prediction model was based on postoperative pathological data and therefore more studies combined preoperative data to further validate our predictive model. Secondly, we were unable to gain preoperative neoadjuvant therapy as suggested, as this study was retrospective and obtained from public data records. However, this did not affect our ability to generate a prediction model of LNM which can guide the subsequent treatment patients, because preoperative treatment affected TS as well as LNM. Lastly, in this study we only explored the effect of TS on LNM and did not combine TNM stage with TS. Therefore, multicenter data studies are needed to validate the results of this study.

## Conclusion

We found that LNM increased with larger TS up to 4 cm in stage T1-T3 Siewert type II AEG patients. The Nomogram presented in this paper provides a simple tool for predicting LNM.

## Figures and Tables

**Table 1:** Baseline characteristics in SEER data

Variable	Total (%)	NLNM (%)	LNM (%)	p	LNMP	mELNc [IQR]	mPLNc [IQR]	mLNRc [IQR]
Total	4649	2246	2403			15 9, 22	1 [0, 3]	0.04 [0.00, 0.24]
**Race**				0.011				
White	4093 (88.0)	2000 (89.0)	2093 (87.1)		51.1	15 9, 22	1 [0, 3]	0.03 [0.00, 0.23]
Black	209 (4.5)	80 (3.6)	129 (5.4)		61.7	16 10, 25	1 [0, 5]	0.09 [0.00, 0.35]
Other	347 (7.5)	166 (7.4)	181 (7.5)		52.2	16 10, 24	1 [0, 4]	0.04 [0.00, 0.25]
**Sex**				0.011				
Male	3738 (80.4)	1771 (78.9)	1967 (81.9)		52.6	15 9, 22	1 [0, 3]	0.04 [0.00, 0.25]
Female	911 (19.6)	475 (21.1)	436 (18.1)		47.9	15 9, 23	0 [0, 3]	0.00 [0.00, 0.21]
**Grade**				<0.001				
G1	292 (6.3)	229 (10.2)	63 (2.6)		21.6	14 [7.75, 20]	0 [0, 0]	0.00 [0.00, 0.00]
G2	1727 (37.1)	996 (44.3)	731 (30.4)		42.3	15 9, 22	0 [0, 2]	0.00 [0.00, 0.15]
G (3+4)	2630 (56.6)	1021 (45.5)	1609 (67.0)		61.2	15 10, 23	1 [0, 4]	0.08 [0.00, 0.33]
**TS team**				<0.001				
1 (0-1 cm)	369 (7.9)	312 (13.9)	57 (2.4)		15.4	12 8, 19	0 [0, 0]	0.00 [0.00, 0.00]
2 (1.1-2 cm)	724 (15.6)	503 (22.4)	221 (9.2)		30.5	13 7, 20	0 [0, 1]	0.00 [0.00, 0.07]
3 (2.1-3 cm)	880 (18.9)	474 (21.1)	406 (16.9)		46.1	15 9, 21	0 [0, 2]	0.00 [0.00, 0.14]
4 (3.1-4 cm)	819 (17.6)	347 (15.4)	472 (19.6)		57.6	15 10, 22	1 [0, 3]	0.07 [0.00, 0.25]
5 (4.1-5 cm	665 (14.3)	222 (9.9)	443 (18.4)		66.6	15 10, 24	2 [0, 5]	0.12 [0.00, 0.34]
6 (5.1-6 cm)	467 (10.0)	172 (7.7)	295 (12.3)		63.2	16 11, 23	2 [0, 5]	0.10 [0.00, 0.34]
7 (6.1-7 cm)	301 (6.5)	95 (4.2)	206 (8.6)		68.4	17 11, 26	2 [0, 7]	0.14 [0.00, 0.41]
8 (≥7.1 cm)	424 (9.1)	121 (5.4)	303 (12.6)		71.5	19 12, 26	3 [0, 7]	0.17 [0.00, 0.50]
**T stage**				<0.001				
T1	1076 (23.1)	852 (37.9)	224 (9.3)		20.8	14 8, 21	0 [0, 0]	0.00 [0.00, 0.00]
T2	1633 (35.1)	642 (28.6)	991 (41.2)		60.7	15 9, 22	1 [0, 4]	0.08 [0.00, 0.30]
T3	1940 (41.7)	752 (33.5)	1188 (49.4)		61.2	16 10, 23	1 [0, 4]	0.08 [0.00, 0.32]
**Age (Year)**				<0.001				
20-40	98 (2.1)	34 (1.5)	64 (2.7)		65.3	17 10, 24	2 [0, 7]	0.12 [0.00, 0.36]
41-50	336 (7.2)	134 (6.0)	202 (8.4)		60.1	15 10, 24	1 [0, 4]	0.09 [0.00, 0.27]
51-60	998 (21.5)	455 (20.3)	543 (22.6)		54.4	15 10, 22	1 [0, 4]	0.05 [0.00, 0.28]
61-70	1546 (33.3)	780 (34.7)	766 (31.9)		49.5	15 10, 22	0 [0, 3]	0.00 [0.00, 0.21]
71-80	1299 (27.9)	666 (29.7)	633 (26.3)		48.7	15 9, 23	0 [0, 3]	0.00 [0.00, 0.20]
≥81	372 (8.0)	177 (7.9)	195 (8.1)		52.4	14 8, 22	1 [0, 3]	0.04 [0.00, 0.25]

TS team, tumor size team; NLNM: no lymph node metastasis; LNM, lymph node metastasis; p, p-value; LNMP, Lymph node metastasis percentage (LNMP (%) = LNM/total LNM*100%); IQR=interquartile range; mELNc, median examined lymph node count (ELN was defined as the number of examined lymph node); mPLNc, meidan positive lymph node count (PLN was defined as the number of positive lymph node); mLNRc, median lymph nodes ratio count (LNR was defined as the ratio between the number of PLN and the total number of ELN).

**Table 2:** Lymph node metastasis or overall survival versus tumor size

Variable	Model^a^		Model^b^		Model^c^	
	HR (95%CI)	P value	OR (95%CI)	P value	OR (95%CI)	P value
**TS team^a^**						
1 (0-1 cm)	1		1		1	
2 (1.1-2 cm)	1.148 (0.940-1.401)	0.176	1.645 (1.174-2.330)	0.004	1.660 (1.183-2.355)	0.004
3 (2.1-3 cm)	1.184 (0.972-1.441)	0.094	2.552 (1.840-3.582)	0.001	2.522 (1.815-3.548)	0.001
4 (3.1-4 cm)	1.378 (1.127-1.685)	0.001	3.544 (2.541-5.004)	0.001	3.480 (2.490-4.921)	0.001
5 (4.1-5 cm)	1.432 (1.165-1.760)	0.001	4.692 (3.325-6.700)	0.001	4.585 (3.243-6.560)	0.001
6 (5.1-6 cm)	1.429 (1.153-1.770)	0.001	4.263 (2.979-6.169)	0.001	4.095 (2.856-5.937)	0.001
7 (6.1-7 cm)	1.686 (1.343-2.116)	0.001	5.086 (3.437-7.612)	0.001	4.798 (3.234-7.198)	0.001
8 (≥7.1 cm)	1.522 (1.224-1.892)	0.001	5.481 (3.776-8.046)	0.001	5.139 (3.532-7.560)	0.001
**P for trend**				0.001		0.001
Tumor size^b^	1.040 (1.026-1.054)	0.001	1.163 (1.129-1.199)	0.001	1.152 (1.118-1.188)	0.001

**Model^a^:** Multivariable Cox regression model for adjustment examined lymph node, age, sex, race, T stage and grade. **Model^b^:** Multivariable logistic regression model for adjustment age, sex, race, T stage and grade. **Model^c^:** Multivariable logistic regression model for adjustment examined lymph node, age, sex, race, T stage and grade. **TS team^a^:** Tumor size as categorical variable. **Tumor size^b^:** Tumor size as continuous variable.

**Table 3:** Baseline characteristics in single-center data

Variable	Cases	Percent (%)	LNMP (%)
**Sex**			
Male	84	77.1	70.2
Female	25	22.9	76
**Grade**			
G (1+2)	77	70.6	64.9
G (3+4)	32	29.4	87.5
**TS team**			
1 (0-1 cm)	5	4.6	0
2 (1.1-2 cm)	7	6.4	57.1
3 (2.1-3 cm)	25	22.9	64
4 (3.1-4 cm)	30	27.5	76.7
5 (4.1-5 cm)	19	17.4	73.7
6 (5.1-6 cm)	12	11	91.7
7 (6.1-7 cm)	5	4.6	100
8 (≥7.1 cm)	6	5.5	83.3
**T stage**			
T1	9	8.3	33.3
T2	18	16.5	33.3
T3	82	75.2	84.1
**Age (Year)**			
20-60	40	36.8	92.5
61-70	41	37.6	58.5
71-80	24	22	58.3
≥81	4	3.7	75

LNMP, Lymph node metastasis percentage; TS team, tumor size team.
